# Music impacts brain cortical microstructural maturation in very preterm infants: A longitudinal diffusion MR imaging study

**DOI:** 10.1016/j.dcn.2023.101254

**Published:** 2023-05-11

**Authors:** Joana Sa de Almeida, Olivier Baud, Sebastien Fau, Francisca Barcos-Munoz, Sebastien Courvoisier, Lara Lordier, François Lazeyras, Petra S. Hüppi

**Affiliations:** a Division of Development and Growth, Department of Paediatrics, Gynecology and Obstetrics, University Hospitals of Geneva, Geneva, Switzerland; b Division of Neonatal and Intensive Care, Department of Paediatrics, Gynecology and Obstetrics, University Hospitals of Geneva, Geneva, Switzerland; c Center of BioMedical Imaging (CIBM), University of Geneva, Geneva, Switzerland; d Department of Radiology and Medical Informatics, Geneva, Switzerland

**Keywords:** Diffusion MRI, Fixel-based analysis, Human preterm brain development, Music intervention, NODDI

## Abstract

Preterm birth disrupts important neurodevelopmental processes occurring from mid-fetal to term-age. Musicotherapy, by enriching infants’ sensory input, might enhance brain maturation during this critical period of activity-dependent plasticity. To study the impact of music on preterm infants’ brain structural changes, we recruited 54 very preterm infants randomized to receive or not a daily music intervention, that have undergone a longitudinal multi-shell diffusion MRI acquisition, before the intervention (at 33 weeks’ gestational age) and after it (at term-equivalent-age). Using whole-brain fixel-based (FBA) and NODDI analysis (n = 40), we showed a longitudinal increase of fiber cross-section (FC) and fiber density (FD) in all major cerebral white matter fibers. Regarding cortical grey matter, FD decreased while FC and orientation dispersion index (ODI) increased, reflecting intracortical multidirectional complexification and intracortical myelination. The music intervention resulted in a significantly higher longitudinal increase of FC and ODI in cortical paralimbic regions, namely the insulo-orbito-temporopolar complex, precuneus/posterior cingulate gyrus, as well as the auditory association cortex. Our results support a longitudinal early brain macro and microstructural maturation of white and cortical grey matter in preterm infants. The music intervention led to an increased intracortical complexity in regions important for socio-emotional development, known to be impaired in preterm infants.

## Highlights

• Preterm infants show specific WM and cortical maturation from 33 to 40 weeks GA.
• Fiber density (FD) and cross-section (FC) increase in WM, indicating myelination.
• In the cortex, FD decreases while FC/ODI increase, suggesting dendritic complexity.
• Music enhances maturation of cortical regions implied in socio-emotional processing.

## 1 Introduction

Prenatal brain development is characterized by important macro- and microstructural changes, in particular during the third trimester, with axonal and dendritic growth, synaptogenesis, myelination and cortical gyration (Knickmeyer et al., 2008, Volpe, 2001). Altogether, these processes lead to an expansion of both grey matter (GM) and white matter (WM) volumes (Huppi et al., 1998b) and maturation of the axonal pathways (Kostovic and Jovanov-Milosevic, 2006, Volpe, 2009). These maturational processes are known to be activity-dependent driven and regulated by the early cortical synaptic activity (Kiss et al., 2014).

Preterm birth interferes abruptly with normal brain maturation during this critical period of brain growth (Back and Miller, 2014, Duvanel et al., 1999, Simmons et al., 2010), potentially leading to various structural brain alterations (Anjari et al., 2007, Ball et al., 2012, Huppi et al., 1998a, Inder et al., 2005, Thompson et al., 2007, Thompson et al., 2013), which have been associated with subsequent neurodevelopmental impairments observed in preterm born infants (Cismaru et al., 2016, Gui et al., 2019, Montagna and Nosarti, 2016; Van't Hooft et al., 2015).

During neonatal intensive care unit (NICU) stay, preterm infants are exposed routinely to multiple noxious stimuli (Kiss et al., 2014, Vinall et al., 2012) and denied meaningful sensory inputs relevant for activity-dependent plasticity (Kiss et al., 2014, Radley and Morrison, 2005). On the other hand, this period offers a window of opportunity to set-up early postnatal interventions aiming at modulating preterm infants’ sensory input and thus enhancing brain activity-dependent maturation. Musicotherapy, in particular, has been proposed as a meaningful sensory stimulation approach during preterm infants’ NICU stay, a period corresponding to a critical window of auditory brain networks maturation (Graven and Browne, 2008, Kiss et al., 2014, Lasky and Williams, 2005). It is known that music listening triggers rich brain processing comprising both cognitive and emotional neural substrates (Koelsch, 2014, Sarkamo et al., 2013, Zatorre et al., 2009). However, there is still limited knowledge regarding the effects of music interventions on the brain during early development, namely its effects on brain structure (Sa de Almeida et al., 2019, Webb et al., 2015).

Diffusion MRI (dMRI) provides unique information about WM fibers, which are not yet fully myelinated in the premature brain and for which relaxation-based contrast is still inadequate (Hermoye et al., 2006, Huppi et al., 1998a, Neil et al., 1998). Diffusion tensor imaging (DTI) has been the most widely used dMRI analysis approach in the developing brain. Using DTI analysis, it was found that an early music intervention during NICU stay leads to enhanced maturation of the acoustic radiations and of the uncinate fasciculus in preterm infants (Sa de Almeida et al., 2019). Recently, higher-order dMRI models capable of resolving multiple fiber orientations within a voxel have been proposed to overcome some of the limitations of DTI. Fixel-based analysis (FBA) is a recent technique that allows characterization of multiple fiber orientations within a voxel and perform whole-brain comparisons of fiber-specific properties. These properties, the FBA metrics, are thus represented within “fixels”, which refer to single fiber populations within a voxel, and comprise: fiber density (FD), reflecting fibers’ microstructural changes in intra-axonal volume/packing; fiber cross-section (FC), referring to macrostructural changes in the fiber bundle cross-sectional area; and fiber density cross-section (FDC), combining both micro and macrostructural changes (Raffelt et al., 2017). Additionally, multi-compartment dMRI models, such as neurite orientation dispersion and density imaging (NODDI) allow characterization of the diffusion weighted (DW) signal in three compartments (intra-neurite, extra-neurite and free water), providing parameters such as intra-cellular volume fraction (ν_ic_, relating to neurite density index, NDI) and orientation dispersion index (ODI, describing the degree of incoherence in fiber orientations) (Zhang et al., 2012). NODDI thus offers thus additional information about tissue microstructure that correlates with histological measures of neurite geometrical complexity and density in both WM and GM (Grussu et al., 2017).

The current study evaluates the longitudinal impact of an early music intervention in very preterm infants’ brain macro- and microstructural development employing recent higher-order dMRI models, namely FBA and NODDI. Unlike two previous studies (Pannek et al., 2018, Pecheva et al., 2019) that applied FBA to study preterm infants’ brain at term-equivalent age, we set up a longitudinal study to assess preterm infants’ brain early structural development from the 33rd week gestational age (GA) to term-equivalent age and, in addition, to evaluate longitudinally the impact of a music intervention on preterm infant’s early brain structural maturation. Moreover, this is also the first time that the NODDI model is applied to complement and evaluate the microstructural modifications underlying the FBA metrics changes.

We hypothesize that: 1) important macro- and microstructural brain maturational changes occur in very preterm infants brain during early development from 33 weeks GA to term-equivalent age; 2) an early postnatal music intervention given during NICU stay enhances important structural brain maturational changes occurring during this period.

## 2 Materials and methods

### 2.1 Subjects

Research Ethics Committee approval was granted for the study and written parental consent was obtained prior to each infant’s participation.

54 very preterm infants (GA at birth <32 weeks) were recruited at the NICU of the University Hospitals of Geneva (HUG), Switzerland, from 2017 to 2020, as part of a blinded randomized controlled clinical trial entitled “The effect of music on preterm infant’s brain development” (NCT03689725), registered at register.clinicaltrials.gov. Out of 54 recruited very preterm infants, 28 received a music intervention during NICU stay (PTM), whereas 26 were randomly allocated to the control group with no music intervention (PTC). Exclusion criteria for all babies included major brain lesions detected on the MRI, such as high-grade intraventricular hemorrhage (grade III or IV), as well as micro- or macrocephaly, hydrocephaly, leukomalacia and congenital syndromes. Six initially eligible and recruited preterm infants were finally not included in the study due to a posteriori parental refusal (1 PTM, 4 PTC) or diagnosis of genetic syndromes (1 PTM).

Very preterm infants underwent an MRI examination at two time-points: during the 33rd week GA (time point 1) and at term-equivalent age (time point 2). Were excluded from the analysis: infants without the MRI acquisition at both time-points, infants whose MRI protocol acquisition was incomplete at one or both time-points (not comprising a T2-weighted image and/or complete multi-shell diffusion imaging (MSDI) sequence) and infants whose images presented registration issues during pre-processing. The final sample used for the analysis consisted of 40 very preterm infants, from which 21 PTM and 19 PTC. The flow chart of participants’ inclusion process is provided in Fig. S1. No significant differences between the PTM and PTC groups were found in demographic and perinatal variables: GA at birth, sex, extremely premature birth, birth weight, birth height, birth head circumference, APGAR at 1 and 5 minutes, intrauterine growth restriction, neonatal asphyxia, bronchopulmonary dysplasia, intraventricular hemorrhage, sepsis (positive blood culture), GA at MRI time-point 1 (33rd week GA), GA at MRI time-point 2 (term-equivalent age), number of music/no music plays and socio-economic parental status score (SES) (Table 1). SES rating was based on recorded mother's education and father's occupation. The scores are distributed from 2 to 12, with 2 indicating higher SES (Largo et al., 1989).

**Table 1:** Clinical characteristics of very preterm infants final sample.

Clinical Characteristics	Preterm Music (PTM) n = 21	Preterm Control (PTC) n = 19	p-value^a^ PTM vs PTC
Gestational age at birth, weeks, mean (SD)	29.40 (±1.7)	28.83 (±2.6)	0.404
Gestational age at birth, weeks, range	27^2/7^ – 32^4/7^	24^1/7^ – 31^6/7^	
Sex: female (%)/male (%)	9(42.8)/12(57.2)	13(68.4)/6(31.6)	0.125
Extremely Premature (<27 weeks GA), n (%)	1 (4.8)	2 (10.5)	0.596
Birth weight, gram, mean (SD)	1241.5 (±450.6)	1129.7 (±328.5)	0.633
Birth height, centimetre, mean (SD)	38.2 (±4.2)	36.8 (±3.2)	0.461
Birth head circumference (cm), mean (SD)	26.3 (±2.6)	26.5 (±3.1)	0.404
APGAR score, 1 min (SD)	5.14 (±3.0)	4.93 (±3.4)	0.900
APGAR score, 5 min (SD)	7.86 (±1.8)	6.73 (±2.2)	0.625
Intrauterine Growth Restriction, n (%)	3 (14.3)	2 (10.5)	1.000
Neonatal asphyxia, n (%)	0	0	
Bronchopulmonary dysplasia, n (%)	8 (38.1)	7 (36.8)	1.000
Sepsis, n (%)	3 (14.3)	6 (31.6)	0.265
Intraventricular Haemorrhage (grade 1), n (%)	4 (19.0)	3 (15.8)	1.000
Gestational age at MRI scan1, weeks, mean (SD)	33.6 (±0.44)	33.7 (±0.43)	0.512
Gestational age at MRI scan1, weeks, range	33^0/7^ – 34^3/7^	32^6/7^– 34^3/7^	
Gestational age at MRI scan2, weeks, mean (SD)	40.1 (±0.58)	40.3 (±0.44)	0.416
Gestational age at MRI scan2, weeks, range	38^5/7^ – 41^1/7^	39^3/7^– 40^6/7^	
Number of music/no music plays	60.52 (±24.2)	55.11 (±28.8)	0.528
Socio-economic score, mean (SD)	3.67 (±2.0)	5.42 (±3.4)	0.051

a Group-characteristics were compared using independent samples t-test for continuous variables and chi-squared test for categorical variables.

### 2.2 Music intervention

Very preterm infants were randomly allocated to either the PTM or the PTC group. The research assistant, clinical staff and parents were blind to the group assignment.

The music intervention was conducted for all very preterm infants from the 33rd week of GA, immediately after the first MRI (time-point 1), until term-equivalent age or until hospital discharge, before the second MRI at term-equivalent age (time-point 2). Infants were exposed to musical pieces approximately two times per day through headphones specifically designed for the study and adapted to preterm infants’ head size (Fig. S2). The control group was equipped identically and received an equivalent number of interventions, but without music listening.

The musical pieces used in this intervention were produced by Andreas Vollenweider (http://vollenweider.com/en), specifically for this project, and are composed of a calming background, bells, harp, and punji (charming snake flute) interactively creating a melody. The musical instruments and melody were chosen based on the behavioral and physiological responses of preterm newborns (for more details consult Appendix B of Lordier et al., 2018). Three different music tracks, of 8 min’ duration, were selected by the nurse according to the state of wakefulness of the child (waking up, falling asleep, being alert). Readiness for the intervention as well as choice of music track was determined based on a neonatal behavioral assessment scale performed bedside by the nurse (Martinet et al., 2013). The 8 min’ duration was chosen to suit the duration of the sleep-wake state transitions of the infant.

### 2.3 MRI acquisition

All infants were scanned after receiving breast or formula feeding during natural sleep (no sedation used). At 33 weeks GA (time-point 1), preterm infants were scanned using a MR compatible incubator (Lammers Medical Technology, Lübeck, Germany) and monitored using a Philips MR patient monitor Expression MR400 and Philips quadtrode MRI compatible neonatal ECG electrodes. At term-equivalent age (time-point 2), infants were scanned using a vacuum mattress for immobilization. All infants, at both time points, were monitored for heart rate and oxygen saturation during the entire scanning time. MR-compatible headphones (MR confon, Magdeburg, Germany) were used to protect the infants from scanner noise.

MRI acquisition at both time-points was performed on a 3.0 T Siemens MR scanner Siemens Magnetom (Siemens, Erlangen, Germany), using a 16-channel neonatal head coil. T2-weighted images were acquired using the following parameters: 113 coronal slices, TR= 4990 ms, TE= 160 ms, flip angle= 150°, matrix size= 256 × 164; voxel size= 0.8 × 0.8 × 1.2 mm^3^. Multi-shell diffusion imaging (MSDI) was acquired with a single-shot spin echo echo-planar imaging (SE-EPI) Stejksal-Tanner sequence (TE=85 ms, TR=3170 ms, voxel size 1.8 ×1.8 ×1.8 mm^3^, multi-band acceleration factor of 2, GRAPPA 2). Images were acquired in the axial plane, in anterior-posterior (AP) phase encoding (PE) direction, with 4 volumes without diffusion-weighting (b0); 10 non-collinear directions with b= 200 s/mm^2^, 30 non-collinear directions with b= 1000 s/mm^2^; 50 non-collinear directions with b= 2000 s/mm^2^. The b0 images were additionally collected with reversed phase-encode blips, AP and posterior-anterior (PA), resulting in pairs of images with distortions going in opposite directions.

### 2.4 Pre-processing

MSDI data were preprocessed using MRtrix3 (version 3.0rc3, https://www.mrtrix.org) (Tournier et al., 2019) for denoising, Gibbs ringing removal, bias field corrections and intensity normalization. FSL diffusion toolbox (v5.0.11, https://fsl.fmrib.ox.ac.uk/fsl/fslwiki/) (Behrens et al., 2003, Smith et al., 2004) was used for estimation of the off-resonance field and correction of susceptibility- and eddy current-induced distortions, as well as subject movement (Andersson and Sotiropoulos, 2016). FSL's TOPUP (Andersson et al., 2003, Smith et al., 2004) was used to estimate the off-resonance field, which was then used as input for FSL's EDDY function optimized for neonatal diffusion data, correcting for distortions induced by susceptibility and eddy currents, as well as by motion-induced signal dropout and intra-volume subject movement (Andersson et al., 2016, Andersson et al., 2017, Bastiani et al., 2019). Data were visually inspected to assure quality of motion artifacts correction.

### 2.5 Fixel-based analysis

After the pre-processing, dMRI images were analyzed using the recommended multi-tissue FBA analysis pipeline from Mrtrix3 (version 3.0rc3).

Each subject data was up-sampled to 1.3 mm^3^ isotropic voxel size and fiber orientation distribution (FOD) was estimated in each voxel using 3-tissue constrained spherical deconvolution (CSD), with group averaged response functions for WM, GM and cerebrospinal fluid (CSF). FOD maps were computed for each subject using a group-average response function (Dhollander et al., 2016).

FBA was performed to study the longitudinal micro- and macrostructural changes occurring during very preterm infants’ brain development, from the 33rd week GA to term-equivalent age, and to evaluate the impact of the music intervention on the significant brain structural changes occurring during this period.

For this purpose, an unbiased study-specific FOD template was computed using 40 intra-subject templates (19 PTC and 21 PTM). In order to generate the intra-subject templates, the time-point 1 and time-point 2 subjects’ FOD maps were first rigidly transformed to midway space and then averaged. These intra-subject templates served as input to create the final study-specific population template used for the longitudinal analyses (Fig. S3). Each subject FOD map, at each time-point, was registered to the final study-specific template. The transformed FOD were segmented to produce discrete fixels, whose directions were re-oriented, and a correspondence to the template space was established, followed by calculation of the FBA metrics (FD, log(FC), and FDC) (Raffelt et al., 2017). Measures of FC were log-transformed prior to statistical analysis (Log(FC)), to ensure that data was centered around zero and normally distributed (Raffelt et al., 2017). FDC is the product of FD and FC (Raffelt et al., 2017).

Whole-brain tractography was performed in template space, using Mrtrix3 iFOD2 algorithm (Tournier et al., 2010), generating 20 million tracts that were subsequently filtered to 2 million tracts using spherical-deconvolution informed filtering of tractograms (SIFT) (Smith et al., 2013).

### 2.6 NODDI analysis

In order to complement the FBA analysis, by further evaluating the microstructural modifications underlying the FBA metrics changes, MSDI data were also analyzed using the neurite orientation dispersion and density imaging (NODDI) model (Zhang et al., 2012). NODDI describes the dMRI signal as a summation of three different compartments: intra-cellular (neurites, restricted), extra-cellular (space around neurites, hindered) and CSF (isotropic). It estimates as parameters 1) the neurite density index (NDI) as the intra-cellular volume fraction (ν_ic_); 2) an orientation dispersion index (ODI) to account for fiber bending and fanning; and also 3) the isotropic volume fraction (ν_iso_). We used Watson distribution to model the distribution of fiber orientations using a GPU-based library for accelerated nonlinear optimization, cuDIMOT (Hernandez Fernandez et al., 2017, www.fmrib.ox.ac.uk/∼moisesf/cudimot) and generate subjects’ ODI and ν_ic_ maps.

### 2.7 Statistical analysis

#### 2.7.1 Whole brain fixel-based analysis

Statistical analysis to evaluate FBA whole-brain structural maturational changes occurring in very preterm infant’s brain from the 33rd week GA to term-equivalent age was performed using a connectivity-based fixel enhancement (CFE) method (Raffelt et al., 2015) with 5000 permutations and family-wise error (FWE) for multiple comparisons correction (statistical significance is reported at p_FWE_ < 0.05). All comparisons were adjusted for GA at MRI at time-point 1, delta of the GA at MRI between the two time-points and sex.

Significant fixels (FWE-corrected p-value < 0.05) were displayed using the *mrview* tool in MRtrix3 on the template-derived tractogram, in which streamlines were cropped to display only significant fixels. Significant streamlines were color-coded either by streamline orientation (left-right: red, inferior-superior: blue, anterior-posterior: green) or by the relative effect size (expressed as a percentage relative to the first time-point). Whole-brain fixel-based statistical analyses and visualizations were performed using MRtrix3.

#### 2.7.2 ROI fixel-based analysis

We aimed to evaluate the effect of the early music intervention on those brain regions found to undergo significant maturational changes in very preterm infants from 33 weeks GA to term-equivalent age. For this purpose, the brain regions containing fixels where the FBA output metrics were significantly different (p_FWE_ < 0.05) between the two time-points were anatomically identified and manually segmented into ROIs, according to DTI atlases (Akazawa et al., 2016, Oishi, 2010). The ROIs were subsequently converted to fixel maps and the FBA output metrics were calculated using these masks for each participant at each time-point. Multivariate repeated measures ANCOVA, controlling for GA at MRI at time-point 1, delta of the GA at MRI between the two time-points and sex was conducted to determine statistically significant differences between PTM and PTC groups on FBA metrics from the 33rd week GA to term-equivalent age (time*group interaction) in the selected brain ROIs, using IBM SPSS Statistics version 25 (IBM Corp., Armonk, N.Y., USA). Given that our data lack sphericity (Mauchly’s sphericity test p < 0.05), we present the p-value results using Greenhouse-Geisser correction. Since the socio-economic parental status score (SES) was marginally significantly different between groups, we present additionally the results of a sensitivity analysis, where we include SES in our model as a covariate. GA at birth was not a significant predictor for any of the dependent variables, which is why it was not included as a covariate in the final analysis.

#### 2.7.3 ROI NODDI analysis

A complementary NODDI analysis was performed to better understand the microstructural alterations underlying the FBA metrics changes observed from the 33rd week GA to term-equivalent age and, in particular, in relation to the music intervention.

The ROIs showing a significantly different longitudinal FBA metrics evolution between PTM and PTC groups were selected and warped to the subject space, using the warps derived from the FBA pipeline. The warped ROIs were used to calculate NODDI metrics in the subjects’ ODI and NDI maps. Multivariate repeated measures ANCOVA, controlling for GA at MRI time-point 1, delta of the GA at MRI between the two time-points and sex was conducted to determine statistically significant differences between PTM and PTC groups on the NODDI metrics from the 33rd week GA to term-equivalent age (time*group interaction) in the selected brain ROIs, using IBM SPSS Statistics version 25 (IBM Corp., Armonk, N.Y., USA). Given that our data lack sphericity (Mauchly’s sphericity test p < 0.05), we present the p-value results using Greenhouse-Geisser correction. Since the socio-economic parental status score (SES) was marginally significantly different between groups, we present additionally the results of a sensitivity analysis, where we include SES in our model as a covariate. GA at birth was not a significant predictor for any of the dependent variables, which is why it was not included as a covariate in the final analysis.

#### 2.7.4 Demographic and perinatal data

To test for differences in demographic and perinatal data between PTC and PTM groups, categorical variables (sex, intrauterine growth restriction, bronchopulmonary dysplasia, intraventricular hemorrhage, blood culture positive sepsis and extremely premature birth) were analyzed using chi-squared test, whereas continuous variables were compared using independent samples *t*-test, with group as independent variable and the following variables as dependent: GA at birth, GA at MRI time point 1, GA at MRI time point 2, birth weight, birth height, birth head circumference, APGAR score at 1 and 5 min after birth and socio-economic parental status score (SES).

### 2.8 Data and code availability

All data were acquired in the context of a research project approved by the ethical committee in 2016. The patient consent form did not include any clause for reuse or sharing of data. Software and code used in this study are publicly available as part of FSL v5.0.10 (https://fsl.fmrib.ox.ac.uk/fsl/fslwiki/), MRtrix3 (Tournier et al., 2019) and cuDIMOT (Hernandez Fernandez et al., 2017, www.fmrib.ox.ac.uk/∼moisesf/cudimot) software packages. dMRI data were pre-processed using EDDY command adapted for neonatal motion, which is part from the neonatal dMRI automated pipeline from developing Human Connectome Project (dHCP, http://www.developingconnectome.org), and can be found at this link: https://git.fmrib.ox.ac.uk/matteob/dHCP_neo_dMRI_pipeline_release (Bastiani et al., 2019).

## 3 Results

### 3.1 Longitudinal whole-brain maturational changes during early development

#### 3.1.1 Preterm brain WM maturational changes from 33 weeks GA to term-equivalent age

**Fig. 1:**
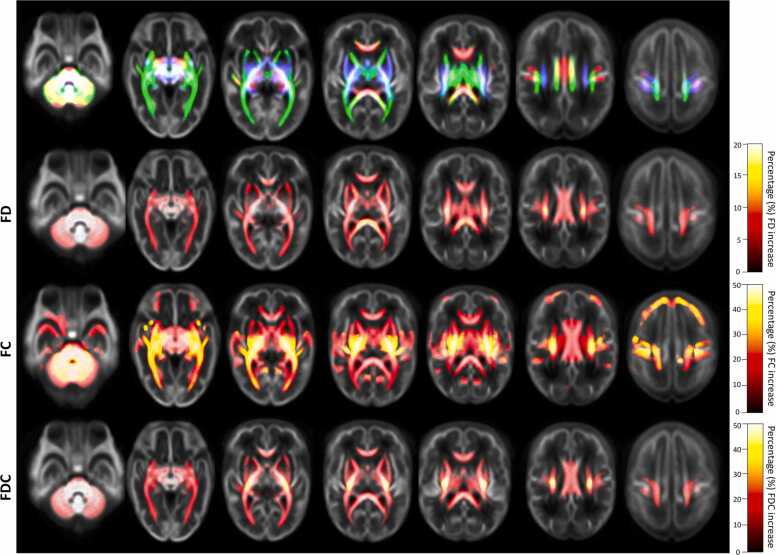
WM fibers with increased FD, FC and FDC from the 33rd week GA to term-equivalent age in very preterm infants’ brain. Population template axial views, from inferior to superior (left to right), of the streamline segments cropped from the template tractogram to include only streamline points that correspond to significant fixels (p_FWE_ < 0.05). Streamlines were colored by direction (anterior-posterior: green, superior-inferior: blue, left-right: red) and percentage of relative effect size increase at term-equivalent age compared to the 33rd week GA, for FD, FC and FDC. From the 33rd week GA to term-equivalent age, there is a statistically significant increase of FD, FC and FDC in all major cerebral WM fibers (commissural, association and projection tracts) bilaterally, as well as in the thalamus, brainstem, cerebellum and cerebellar peduncles.

In the preterm brain, from the 33rd week GA to term-equivalent age we find a statistically significant (p_FWE_ < 0.05) longitudinal increase of FD, FC and FDC in all major brain WM fibers, comprising commissural fibers: body, splenium and genu of corpus callosum, forceps major, forceps minor, anterior commissure; as well as association fibers bilaterally: cingulum, superior longitudinal fasciculus, arcuate fasciculus, inferior fronto-occipital fasciculus, inferior longitudinal fasciculus, uncinate fasciculus; and projection fibers bilaterally: superior corona radiata, fibers passing in the internal capsule (such as the cortico-spinal tract) and in the external capsule, as well as the fornix, optic and acoustic radiations. Additionally, there was also a significant increase of FD, FC and FDC in the thalamus (from where the main ascending projection fibers originate), in the brainstem (where projection fibers pass) and in the cerebellum and cerebellar peduncles (Fig. 1). In addition to these WM changes, we have also observed an increase of FC in certain cortical GM regions, which will be further specified in the following Section 3.1.2.

Whole-brain macrostructural increases of FC were generally more pronounced (until 50% of increase) than microstructural FD increases (until 20% of increase). The WM fibers with the highest FD, FC and FDC increase from the 33rd week GA to term-equivalent age were the projection fibers (Fig. 1).

#### 3.1.2 Preterm brain cortical GM maturational changes from the 33rd week GA to term-equivalent age

**Fig. 2:**
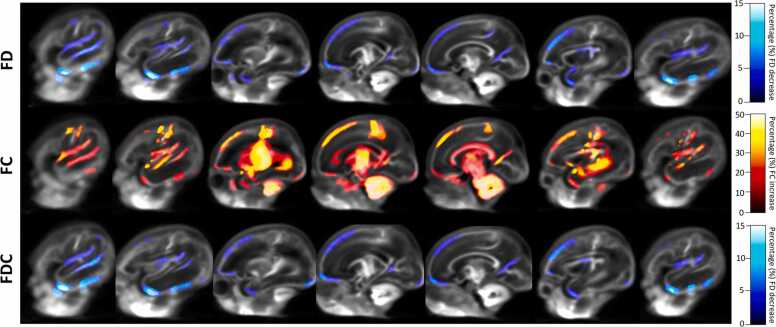
Cortical GM regions with increased FC and decreased FD and FDC from the 33rd week GA to term-equivalent age in very preterm infants’ brain. Population template sagittal views, from the right to left hemisphere, including only points that correspond to significant fixels (p_FWE_ < 0.05). Streamlines were colored by percentage or relative effect size increase at term-equivalent age in comparison to the 33rd week GA for FD, FC and FDC. From the 33rd week GA to term-equivalent age there was an increase of FC (images colored in red) and decrease of FD and FDC (images colored in blue) in the following cortical GM areas: bilateral orbito-frontal cortex, dorsal superior frontal gyrus, medial frontal gyrus, precentral cortex, postcentral cortex, Heschl's gyrus, superior and inferior temporal gyrus, right middle temporal gyrus, bilateral insula, precuneus/posterior cingulate gyrus, calcarine gyrus, lingual gyrus and inferior occipital gyrus.

In the preterm brain, from the 33rd week GA to term-equivalent age we find a statistically significant longitudinal increase of FC and decrease of FD and FDC (p_FWE_ < 0.05) in the following cortical GM regions: bilateral orbito-frontal cortex, dorsal superior frontal gyrus, medial frontal gyrus, precentral cortex, postcentral cortex, Heschl's gyrus, superior and inferior temporal gyrus, right middle temporal gyrus, bilateral insula, precuneus/posterior cingulate gyrus, calcarine sulcus, lingual gyrus and inferior occipital gyrus (Fig. 2).

During this period of early brain maturation, cortical GM macrostructural increases of FC were pronounced (up to 50% of increase) and accompanied by a decrease in FD and FDC by up to 15% in the same cortical regions. The largest FC increase occurred in the bilateral dorsal superior frontal gyrus, precentral and postcentral cortex, Heschl's gyrus, superior temporal gyrus, insula and precuneus/posterior cingulate gyrus, whereas the largest FD and FDC decrease occurred in the bilateral temporal pole, inferior temporal gyrus, bilateral orbito-frontal cortex and left dorsal superior frontal gyrus.

### 3.2 Effect of music on longitudinal brain maturation during early development

#### 3.2.1 Effect of music on WM fibers FBA metrics

We aimed to evaluate the effect of music on those brain regions found to undergo important maturational changes from 33 weeks GA to term-equivalent age. For that, fixel masks corresponding to the major WM tracts with a significant (pFWE < 0.05) longitudinal FD, FC and FDC increase were used for computing FBA metrics longitudinal changes. The fixel masks used comprised (as described in Section 3.1.1.): the corpus callosum (body, genu and splenium), forceps major, forceps minor, anterior commissure and, bilaterally, the cingulum, superior longitudinal fasciculus, inferior fronto-occipital fasciculus/inferior longitudinal fasciculus, cortico-spinal tract, external capsule, uncinate fasciculus, fornix, acoustic radiations and optic radiations. There were no significant statistical differences between PTM and PTC infants regarding FBA metrics longitudinal changes on the selected tracts (p > 0.05).

#### 3.2.2 Effect of music on cortical GM FBA metrics

In addition to the WM, we aimed to evaluate the effect of the music intervention on the cortical GM regions undergoing significant longitudinal maturational changes. For that, fixel masks containing the cortical regions showing a significant FC increase and FD and FDC decreases, from the 33rd week GA to term-equivalent age, were used for computing FBA metrics longitudinal changes. The fixel masks used comprised (as described in Section 3.1.2.): left and right: orbito-frontal cortex, medial frontal gyrus, dorsal superior frontal gyrus, precentral cortex, postcentral cortex, Heschl's gyrus, superior temporal gyrus, inferior temporal gyrus, precuneus/posterior cingulate gyrus, insula and calcarine/lingual/inferior occipital gyrus; as well as the right middle temporal gyrus.

**Fig. 3:**
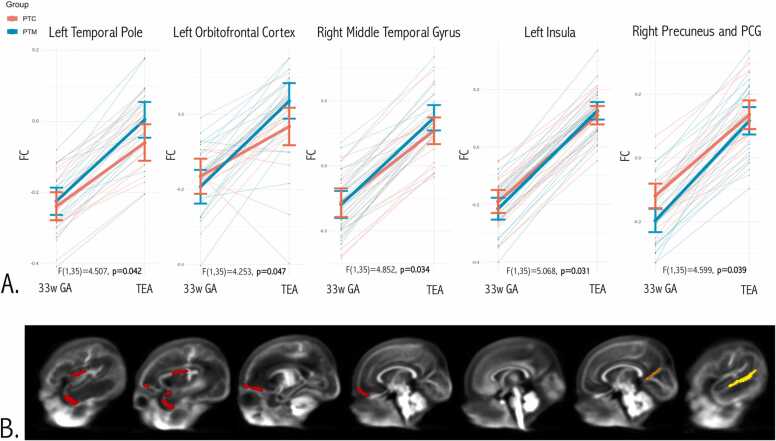
Cortical GM regions with a significantly superior longitudinal increase of FC in PTM in comparison to PTC, from the 33rd week GA to term-equivalent age. A. Longitudinal graph per region - FC values were averaged across the GM cortical masks (left temporal pole, left orbito-frontal cortex, right middle temporal gyrus, left insula and right precuneus/posterior cingulate gyrus) for each subject and plotted against age. Multivariate repeated measures ANCOVA with correction for gestational age at MRI time-point 1, delta of the time between the two MRI and sex was performed. Mean and 95% confidence intervals are illustrated in blue for PTM and in red for PTC infants. B. Population template sagittal views, from the left to right hemisphere, including the cortical brain regions with significant superior longitudinal FC increase in PTM in comparison to PTC – left insulo-orbito-temporopolar complex (red), right precuneus/posterior cingulate gyrus (orange) and right middle temporal gyrus (yellow).

When comparing the FBA metrics longitudinal changes between groups, in comparison to PTC, PTM had a significantly superior FC increase in the left temporal pole (F(1,32)= 4.507, p = 0.042), left orbito-frontal cortex (F(1,35)= 4.253, p = 0.047), right middle temporal gyrus (F(1,35)= 4.852, p = 0.034), left insula (F(1,35)= 5.068, p = 0.031) and right precuneus/posterior cingulate gyrus (F(1,35)= 4.599, p = 0.039) (Fig. 3). No significant differences were found for the remaining cortical regions regarding FBA metrics longitudinal change between groups, (p > 0.05). When performing a sensitivity analysis by including also the socio-economic parental status score (SES) as a covariate in the model, in comparison to PTC, PTM had a significantly superior FC increase in the left temporal pole (p = 0.042), left orbitofrontal cortex (p = 0.047), right middle temporal cortex (p = 0.035) and left insula (p = 0.024), but not anymore in the right precuneus/posterior cingulate gyrus (p = 0.098), although there was a tendency for a greater longitudinal FC increase in this region in the PTM group in comparison to the PTC group.

#### 3.2.3 Effect of music on cortical GM NODDI metrics

A complementary cortical GM NODDI analysis was performed, in order to better understand the observed longitudinal FBA microstructural changes related to the music intervention on the cortical GM. For that, masks containing the cortical regions showing a significantly superior longitudinal increase of FC in PTM in comparison to PTC infants were warped from the population template to the subject space, at each time point. These cortical masks were then used to evaluate the NODDI parameters longitudinal evolution differences between both groups. The masks used comprised (as described in Section 3.2.2.): the left orbito-frontal cortex, left temporal pole, left insula, right middle temporal gyrus and right precuneus/posterior cingulate gyrus.

For both groups, in all cortical regions evaluated, there was a significant ODI increase over time, from the 33rd week GA to term-equivalent age (p < 0.0001).

**Fig. 4:**
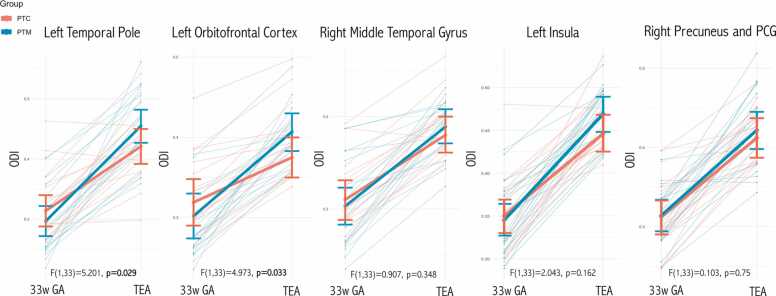
ODI longitudinal changes per group, from the 33rd week GA to term-equivalent age, in the cortical GM regions that had a significant longitudinal FC increase in PTM in comparison to PTC**.** ODI values were averaged across the GM cortical regions masks (left temporal pole, left orbito-frontal cortex, left insula, right middle temporal gyrus and right precuneus/posterior cingulate gyrus) for each subject and plotted against age. In all cortical regions evaluated there was a significant ODI increase from the 33rd week GA to term-equivalent age. In comparison to PTC, PTM had a significantly superior ODI increase in the left temporal pole (p = 0.029) and left orbito-frontal cortex (p = 0.033). Multivariate repeated measures ANCOVA with correction for gestational age at MRI time-point 1, delta of the time between the two MRI and sex was performed. Mean and 95% confidence intervals are illustrated in blue for PTM and red for PTC infants.

When comparing the NODDI metrics longitudinal changes between groups, in comparison to PTC, PTM had a significantly superior ODI increase in the left temporal pole (F(1,33)= 5.201, p = 0.029) and left orbito-frontal cortex (F(1,33)= 4.973, p = 0.033). In the other cortical regions, namely the right middle temporal gyrus, left insula and right precuneus/posterior cingulate gyrus, there was a tendency for a superior ODI longitudinal increase in the PTM vs PTC group, but without a statistically significant difference (Fig. 4).

The additional inclusion of the socio-economic parental status score (SES) as a covariate in the model led to similar results: in comparison to PTC, PTM had a significantly superior ODI increase in left temporal pole (p = 0.021) and left orbitofrontal cortex (p = 0.026); the other three cortical regions remained non-significant (right middle temporal cortex [p = 0.510], left insula [p = 0.167] and right precuneus/posterior cingulate gyrus [p = 0.850]).

No significant differences between groups were found for NDI in all the evaluated cortical regions (p > 0.05).

## 4 Discussion

In this longitudinal study we have evaluated preterm infants’ brain early structural changes, as well as the effect of an early music intervention in these structural changes occurring from the 33rd week GA to term-equivalent age. For that we used FBA, a recent method that allows to investigate whole-brain fiber-specific micro- and macrostructural changes, complemented with NODDI analysis, in order to better understand the observed microstructural changes related to the music intervention.

The major findings from this study are: 1) there is an important longitudinal increase of FD, FDC and especially of FC, in all major cerebral WM fibers bilaterally, as well as in the thalamus, brainstem, cerebellum and cerebellar peduncles, revealing thus important micro- and macroscopic maturation of the WM fiber bundles during this period, suggesting axonal growth and myelination. Interestingly, from all WM fibers, the projection fibers were the ones exhibiting the most prominent maturational changes; 2) in diverse cortical GM regions, there is a significant increase of FC, accompanied by a decrease of FD and FDC, revealing important intracortical maturational changes occurring during this period, characterized by multidirectional complexification as well as intracortical myelination; 3) the music intervention lead to a longitudinal accelerated maturation of specific cortical GM regions, marked by a significantly superior increase of FC in the left insulo-orbito-temporopolar complex, right precuneus/posterior cingulate gyrus and right middle temporal gyrus, accompanied, in addition, of a significantly superior ODI increase in the left temporal pole and left orbito-frontal cortex.

### 4.1 Premature infant’s brain undergoes important micro and macrostructural maturational changes in both WM and GM during early brain development

The longitudinal increase of FD, FC and FDC observed in premature infants’ WM tracts, from the 33rd week GA to term-equivalent age, relates to the important axonal growth, maturation and myelination processes occurring typically during the third trimester of pregnancy and leading to the establishment of long-range WM connections, facilitating brain integration (Batalle et al., 2017, Kostovic and Jovanov-Milosevic, 2006, Kostovic and Judas, 2010, Kostovic et al., 2014, van den Heuvel et al., 2015).

Our FBA results are in line with previous dMRI studies supporting an increased maturation of WM fibers during early brain development, characterized by an increase of NDI and fractional anisotropy (FA), and decrease of ODI and mean diffusivity (MD), relating to increased fiber organization, axonal coherence and preliminary myelination (Aeby et al., 2009, Batalle et al., 2017, Dean et al., 2017, Dubois et al., 2008, Huppi et al., 1998a, Kunz et al., 2014, Mukherjee et al., 2002, Nossin-Manor et al., 2013, Shim et al., 2012). Furthermore, previous studies applying FBA to investigate WM changes occurring in children and adolescents brain development have also shown an increase of FD, FC and FDC in major cerebral WM tracts, supporting a continuous WM maturation throughout childhood (Dimond et al., 2020, Genc et al., 2018, Kelly et al., 2020).

Moreover, the increase of FD, FC and FDC in thalamic regions supports the fact that deep GM also undergoes important maturational processes, which is in line with the rapid growth and establishment of functional thalamo-cortical connections occurring during this critical period of brain development (Batalle et al., 2017, Kostovic and Jovanov-Milosevic, 2006, Kostovic and Judas, 2010, Krsnik et al., 2017, Pouchelon and Jabaudon, 2014).

Our results additionally show that the observed maturation of WM tracts is characterized mainly by an important increase of FC, up to 50%, reflecting thus a predominant macroscopic spatial extent of the fiber bundles, accompanied by an increase of up to 15% of FD, reflecting microstructural axonal number expansion. This greater increase of FC in comparison to FD might be related to maturational processes such as myelination, leading to a prominent increase in fiber bundle diameter and thus increased ability to transfer information across brain regions (Baumann and Pham-Dinh, 2001, Volpe, 2001). Also during early childhood, FC changes in WM were proven to be more widespread and to present a greater percentage increase than changes in FD, supporting that increases in fiber-bundle size might be the major contributor to WM maturation during development (Dimond et al., 2020).

From all WM tracts, the central projection fibers are the ones revealing the highest rates of FD, FC and FDC increase. Such is in line with the expected maturation and myelination trajectory, known to occur primarily in central regions and projection fibers (Kinney et al., 1988). This might be related to the fact that earlier myelination in longer WM bundles might be necessary to compensate for brain growth and maintain similar communication latencies between brain regions (Salami et al., 2003).

In parallel to WM maturation, our study also unveils important maturational changes occurring in cortical GM during this early period of postnatal preterm brain development. A significant increase of FC accompanied by a decrease of FD and FDC is observed in various cortical GM regions distributed among the frontal, parietal, temporal, insula, cingulate and occipital cortices, comprising all the primary sensory-motor areas (auditory, somatosensory, visual and primary motor cortex).

Both FD and FDC are directional-dependent metrics, since FD is computed by integrating the FOD amplitude proportional to the volume of the fibers aligned in a certain direction (Raffelt et al., 2017). Such can explain the decrease in FD and FDC observed in cortical GM, since multiple cellular components with different directions are thought to contribute to the FOD amplitude in GM, namely dendrites, axons and astrocytes processes (Budde et al., 2011, Budde and Annese, 2013). In fact, studies in both human and animal models have shown that the cortical FOD presents an important radial orientation component explained by the apical dendrites (Aggarwal et al., 2015, Assaf, 2019, Budde and Annese, 2013, Dudink et al., 2015, Heidemann et al., 2010, Kleinnijenhuis et al., 2013, Leuze et al., 2014, Sizonenko et al., 2007). However, cortical maturation during the third trimester of pregnancy is characterized by an important increase in dendritic arborisation and geometric complexity, with increased neuronal connections number, proliferation of glial cells, disappearance of the radial glia and its transformation into astrocytes, as well as formation of basal dendrites cross-connections running parallel to the cortical surface, which leads to a more complex multidirectional and less radial environment (Bystron et al., 2008, Cameron and Rakic, 1991, He et al., 2020, Marin-Padilla, 1992, Rakic, 2003). The observed longitudinal decrease of cortical FD and FDC might thus reflect this important cortical geometrical complexification (Bystron et al., 2008, He et al., 2020), similarly to the reported cortical FA decrease (also directionality-dependent) when using DTI to study early cortical maturation (Ball et al., 2013, Batalle et al., 2019, Eaton-Rosen et al., 2015, McKinstry et al., 2002). Furthermore, when applying NODDI, studies show an ODI increase in the cortex (Batalle et al., 2019), equally linked to dendritic arborisation, synaptic formation, myelination of intracortical axons and disruption of the well-organized radial glia scaffold, resulting in a intracortical multidirectional complexity (Assaf, 2019, McKinstry et al., 2002, Smyser et al., 2016). Additionally, the decrease of FD in cortical GM during early brain development might also be related to the cortical expansion occurring during this period (Huttenlocher, 1990), as well as cell death or apoptosis, leading to a reduction of the neuronal density in the cortical plate (Chan and Yew, 1998, Lossi and Merighi, 2003).

On the other hand, the observed significant increase in cortical FC, accompanying FD and FDC decrease, can also be related to myelination of intracortical axons, as well as to the expansion of basal dendrites and glial cells during development. Indeed, a decreased FC has been related to decreased myelination (Gajamange et al., 2018), which has been confirmed by histological staining (Malhotra et al., 2019). Interestingly, the highest FC increase was observed in primary sensorial and motor processing areas, namely precentral cortex, postcentral cortex and Heschl's gyrus, which have been proven to be the first cortical regions to demonstrate the presence of intra-cortical myelin (Huttenlocher and Dabholkar, 1997, Rowley et al., 2017). Furthermore, the fact that the primary motor, somatosensory, visual and auditory cortices are among the regions showing significant cortical maturational changes is in line with the early establishment of activity-dependent thalamo-cortical connectivity, transmitting the environmental extrinsic input through the sensory thalamic nuclei to primary cortical regions, what may contribute to increased synaptogenesis and intra-cortical myelination during this period.

### 4.2 Early music intervention enhances cortical GM maturation during early brain development

Based on the observed brain maturational changes occurring from the 33rd week GA to term-equivalent age, we have further evaluated the longitudinal effect of the music intervention on those maturational processes.

Our results suggest that an early postnatal music intervention, given to very preterm infants during NICU stay, enhances the maturation of certain cortical GM regions. In particular, it led to a FC increase in the left orbito-frontal cortex, left temporal pole, left insula, right middle temporal gyrus and right precuneus/posterior cingulate gyrus. This FC increase possibly reflects an increase in the number of dendrites and intra-cortical myelination. Indeed, the third trimester of pregnancy is marked by an important activity-dependent neural plasticity. The thalamocortical neurons bridge the cortical circuitry with sensory periphery, allowing sensory-driven activity to modulate neuronal connections. Providing an external organized auditory stimulus in the form of music to very preterm infants during this critical period, has thus led to an enhanced cortical maturation of regions important mostly for social-emotional processing.

The orbito-frontal cortex, temporal pole and insula constitute what is known as the insulo-orbito-temporopolar complex of the paralimbic brain, sharing histologic and functional similarities (Peters and Jones, 1985) and being involved in sensory integration, processing of affective stimuli and evaluation of emotional association (Chang et al., 2013, Kringelbach, 2005, Mackes et al., 2018, Olson et al., 2007, Wildgruber et al., 2005). Music listening has been shown to activate all these cortical regions in the human brain (Koelsch, 2010, Koelsch, 2014). Additionally, the auditory cortex has been proven to be functionally connected to these regions when musical stimuli were eliciting emotions (Koelsch et al., 2018).

These findings are in line with our previous study, showing that a similar early postnatal music intervention during NICU stay led to a significant increase of FA in the uncinate fasciculus, a WM bundle connecting the orbito-frontal cortex to the temporal pole, in very preterm infants at term-equivalent age (Sa de Almeida et al., 2019). Additionally, previous functional studies are also in line with the current results. Resting-state functional connectivity was studied at term-equivalent age in very preterm infants that were exposed to a similar musical intervention, showing that it led to a significantly higher functional connectivity between the salience network, in which the insula is a main component, with other brain areas, such as the thalamus, precuneus and frontal regions, in comparison to very preterm infants in the control group (Lordier et al., 2019). There is also evidence suggesting that music training could lead to a significant reorganization of insula-based networks, potentially facilitating high-level cognitive and affective functions (Zamorano et al., 2017). Moreover, very preterm infants exposed to the similar music intervention, when re-listening to the music at term-equivalent age during the MRI acquisition, also revealed an increased functional connectivity between the right temporal pole and the left superior parietal gyrus and left thalamus, as well as between the right orbitofrontal region and the right postcentral gyrus, and also between the right amygdala and the right thalamus; regions implicated in music, multisensory and emotional processing (Loukas et al., 2022).

It is important to highlight that structural alterations affecting the insulo-orbito-temporopolar complex have been further related to the later socio-emotional deficits observed in preterm born infants (Aanes et al., 2015, Cismaru et al., 2016, Fischi-Gomez et al., 2015, Nosarti et al., 2014, Peterson et al., 2000, Rogers et al., 2012) and that up to 25% of very preterm infants evidence an impaired behavioral and socio-emotional development (Anderson and Doyle, 2003, Arpi and Ferrari, 2013, Bhutta et al., 2002, Spittle et al., 2009, Witt et al., 2014). A previous study from our group has shown that preterm infants that have participated in a similar music intervention during NICU stay presented fear-reactivity scores at 12-months and anger-reactivity scores at 24-months of age that were closer to full-term newborns, when compared to preterm infants from the control group (Lejeune et al., 2019), supporting a positive long-term impact of the early music intervention in premature infants’ socio-emotional development.

The right middle temporal gyrus is part of the auditory association cortex and has been shown to be activated during passive music listening (Ohnishi et al., 2001) and also when musical stimuli were eliciting emotions (Koelsch et al., 2018). Patients with lesions in the middle temporal cortex were shown to have difficulty in interpreting the emotional tone of prosody (Peretz et al., 1994).

Regarding the precuneus and the posterior cingulate cortex, these regions have been shown (together with the auditory cortex) to be functionally activated when musical stimuli elicit emotions (Koelsch et al., 2018). Furthermore, the precuneus and the posterior cingulate cortex constitute together the posterior part of the “default mode network” (DMN), functionally linked to self-related processes (Cavanna and Trimble, 2006, Fransson and Marrelec, 2008), social cognition (Mars et al., 2012) and inhibitory control (Fair et al., 2008, Whitfield-Gabrieli and Ford, 2012). This posterior component of DMN was shown to have a decreased functional connectivity in the preterm population (Lordier et al., 2019, Smyser et al., 2010, White et al., 2014). As referred above, preterm infants receiving a similar music intervention during NICU stay presented an increased functional connectivity at term-equivalent age between the salience network and the precuneus (Lordier et al., 2019). Adult studies have also shown an increased functional connectivity between the salience network and DMN in response to passive music listening (Sridharan et al., 2008).

When controlling for socio-economic parental status score (SES) in our additional sensitivity analysis, the longitudinal FC increase remains significantly greater in the music intervention group in comparison to the control group for the same brain regions, except for the precuneus/posterior cingulate gyrus. There is actually a tendency for a greater longitudinal FC increase in this region in the music group in comparison to the control group, but this difference is no longer significant. We can thus consider that for this particular region the variability found between groups diminishes when correcting for SES, which is marginally significantly superior in the music group in comparison to the control group. Previous studies have shown that SES is strongly associated with preterm infant’s later cognitive outcomes, namely at 18–24 months and 5 years of age, although not with brain volumes at term-equivalent age (Gui et al., 2018). Since the precuneus/posterior cingulate gyrus complex is known to have an important role in cognition (Buckner et al., 2008, Leech and Sharp, 2014), it is possible that this region maturation might be influenced, already early in life, by the SES.

Another interesting finding is the laterality effect of the music intervention on the right middle temporal gyrus, the left insulo-orbito-temporopolar complex and the right precuneus/posterior cingulate gyrus. In adults, key acoustic characteristics of melodic and harmonic music have been shown to activate right cortical auditory areas (Tervaniemi and Hugdahl, 2003). In newborns, however, there is controversial evidence whether there is a laterality regarding music processing, which remains an open question. There are functional studies showing no significant consistent laterality for music in newborn infants, with both hemispheres being activated by music stimuli (Kotilahti et al., 2010), whereas other studies show that newborns in the first days of life process music stimuli predominantly in the right primary and higher order auditory cortex, with co-activation of the left inferior frontal and limbic structures (Perani et al., 2010), which is in line with our findings. Our study is the first showing a laterality effect of a music intervention on cortical structural maturation in newborns.

Altogether, our findings support that an early postnatal music intervention given to very preterm infants during NICU stay may induce the maturation of specific cortical regions undergoing important maturational changes from the 33rd week GA to term-equivalent age and that have been previously shown be affected by premature birth. These cortical regions comprise important paralimbic cortical areas such as the left insulo-orbito-temporopolar complex, the right posterior component of DMN (right precuneus/posterior cingulate gyrus), as well as the right auditory association cortex, what might contribute to mitigate the deficits in behavior and social-emotional cognition observed later on in the preterm population.

### 4.3 Cortical ODI increase accompanies FC increase after early music intervention

To better evaluate the microstructural modifications underlying the observed cortical FC increase following the early music intervention, we have complemented our FBA with a NODDI analysis, applied specifically to those cortical regions where we observed a significant effect of the music intervention.

In all the evaluated cortical regions exhibiting a significantly superior FC longitudinal increase from the 33rd week GA to term-equivalent age, we also found a significant longitudinal ODI increase, in line with previous results using NODDI to evaluate early cortical maturation (Batalle et al., 2019). Interestingly, this ODI increase is significantly greater for the group that listened to music in the left orbito-frontal cortex and left temporal pole, further supporting an effect of the music intervention on these cortical regions microstructural organisation. These results remain significant after inclusion of SES as a covariate in our sensitivity analysis.

ODI relates to the degree of incoherence of fiber orientations, which has been linked to an increased dendritic arborisation and consequent geometrical complexification in cortical GM (Wang et al., 2019, Zhang et al., 2012). The observed cortical ODI increase from the 33rd week GA to term-equivalent age supports an increase in dendrite arborisation occurring in the cortical plate during this period. During early brain development, it has been shown that ODI increases until term age and then stabilizes, supporting that until term-age cortical maturation is dominated by an increase in dendritic arborisation and complexity, congruent with the observed increase on cortical volume and curvature (Batalle et al., 2019, Eaton-Rosen et al., 2015).

The cortical ODI increase is in line with the increase in cortical FC, since it supports that a higher number of dendritic branches are present when the cross-sectional plan is evaluated; as well as with the parallel FD decrease, given that the complex cortical microstructure may reduce FD, which is a directional-dependent metric.

Our results further support the hypothesis that the early music intervention, by enriching the auditory environment of preterm infants during a critical period of cortical activity-dependent plasticity, might modify the establishment and remodelling of the incoming thalamo-cortical connections and enhance cortical maturation, due to an increase in cortical dendritic arborisation and complexity, in regions known to be structurally impaired by preterm birth and related to the behavior and socio-emotional difficulties observed later in this population (Aanes et al., 2015, Anjari et al., 2007, Ball et al., 2012, Cismaru et al., 2016, Fischi-Gomez et al., 2015, Gimenez et al., 2006, Huppi et al., 1998a, Inder et al., 2005, Nosarti et al., 2014, Peterson et al., 2000, Rogers et al., 2012, Thompson et al., 2007, Thompson et al., 2013).

## 5 Limitations

This study has limitations that have to be considered. First, our sample size is modest for a randomized clinical trial. This limitation is mainly related to a difficulty of recruitment of preterm infants and families into a research project during a stressful time in their life. In addition, this trial has been conducted, in part, during the Sars-Cov-2 pandemic, what has also imposed limitations regarding patients’ recruitment. Second, the number of times of music exposure during NICU stay can also be considered as limited. However, there are still no guidelines regarding the most adequate dosage to be used in music therapy. More studies concerning this question and correlating results with a quantification of music exposure should be ideally performed to better define dose effects. Third, due to diffusion imaging resolution and co-registration of the two images of one subject over time our analysis may be affected by partial volume effects. Nevertheless, since our analysis is automatized, any potential systematic errors would be similar among all subjects, thus allowing for a fair comparison between groups. Forth, the clinical significance behind brain structural alterations, such as FBA and NODDI metrics, following the music intervention, remains to be evaluated by investigating neurodevelopmental and cognitive outcomes in childhood, which are planned in our follow-up studies. Fifth, noise exposure during NICU stay was not quantified in this study and assumed to be equally distributed among groups, given the random allocation process and the similar topology of infants’ single rooms in neonatology. This would be an interesting feature to address in future studies. Finally, this study has compared a recorded instrumental music intervention to a similar condition but with absence of music during NICU stay. Parental presence and exposure to voice, including maternal singing, were considered to be equally distributed between groups, given the process of random allocation, and were not individually quantified. However, it might also hold an impact on preterm infant’s brain development. In general, more research is needed to compare instrumental music interventions to other sound interventions, such as maternal singing.

## 6 Conclusion

Using a longitudinal whole-brain FBA analysis, we have shown that important WM and GM maturational changes take place from the 33rd week GA of preterm brain development to term-equivalent age. In particular, all major cerebral WM fibers undergo significant increases in microscopic fiber density and, specially, in macroscopic fiber bundle cross-section, with the most pronounced changes occurring in the central projection fibers. In the cortical GM, the FBA metrics changes support an important increase in dendrite growth and arborisation, resulting in a complexification of the cortical geometric structure of diverse regions. Furthermore, an early music intervention given to preterm infants during NICU stay has led to an enhanced maturation of cortical regions undergoing important maturational changes from the 33rd week GA to term-equivalent age and important for auditory, cognitive and, in particular, socio-emotional processing. The effect of music on these cortical regions seems to be related to a cortical complexification, possibly in line with increase in dendrite growth and arborisation, as well as intracortical myelination. Early interventions, such as music, aiming to enrich the sensory input given to preterm infants during this important period of activity-dependent brain plasticity, might therefore play a role in these infants later clinical development. In particular, music may activate and modulate cortical regions involved in emotional processing and thus contribute to mitigate the later socio-emotional problems observed in preterm population, assisting preterm infants to achieve its full potential.

## Funding

This study was supported by grants from the 10.13039/501100001711Swiss National Science Foundation (no. 32473B_135817/1 and no. 324730–163084), the Prim'enfance Foundation, foundation ART-THERAPIE, the Swiss Government Excellence Scholarship (no. 2017.0450/OP),the 10.13039/501100008485Swiss Academy of Medical Sciences (YTCR 49/19) and the Fondation pour la recherche en périnatalité.

## Declaration of Competing Interest

The authors declare that they have no known competing financial interests or personal relationships that could have appeared to influence the work reported in this paper.

## Data Availability

The authors do not have permission to share data.

## References

[bib1] Aanes S. (2015). Memory function and hippocampal volumes in preterm born very-low-birth-weight (VLBW) young adults. Neuroimage.

[bib2] Aeby A. (2009). Maturation of thalamic radiations between 34 and 41 weeks' gestation: a combined voxel-based study and probabilistic tractography with diffusion tensor imaging. AJNR Am. J. Neuroradiol..

[bib3] Aggarwal M. (2015). Probing region-specific microstructure of human cortical areas using high angular and spatial resolution diffusion MRI. Neuroimage.

[bib4] Akazawa K. (2016). Probabilistic maps of the white matter tracts with known associated functions on the neonatal brain atlas: Application to evaluate longitudinal developmental trajectories in term-born and preterm-born infants. Neuroimage.

[bib5] Anderson P., Doyle L.W. (2003). Neurobehavioral outcomes of school-age children born extremely low birth weight or very preterm in the 1990s. Jama-J. Am. Med. Assoc..

[bib6] Andersson J.L.R. (2016). Incorporating outlier detection and replacement into a non-parametric framework for movement and distortion correction of diffusion MR images. Neuroimage.

[bib7] Andersson J.L.R. (2017). Towards a comprehensive framework for movement and distortion correction of diffusion MR images: Within volume movement. Neuroimage.

[bib8] Andersson J.L.R., Sotiropoulos S.N. (2016). An integrated approach to correction for off-resonance effects and subject movement in diffusion MR imaging. Neuroimage.

[bib9] Andersson J.L.R., Skare S., Ashburner J. (2003). How to correct susceptibility distortions in spin-echo echo-planar images: application to diffusion tensor imaging. Neuroimage.

[bib10] Anjari M. (2007). Diffusion tensor imaging with tract-based spatial statistics reveals local white matter abnormalities in preterm infants. Neuroimage.

[bib11] Arpi E., Ferrari F. (2013). Preterm birth and behaviour problems in infants and preschool-age children: a review of the recent literature. Dev. Med. Child Neurol..

[bib12] Assaf Y. (2019). Imaging laminar structures in the gray matter with diffusion MRI. Neuroimage.

[bib13] Back S.A., Miller S.P. (2014). Brain injury in premature neonates: a primary cerebral dysmaturation disorder?. Ann. Neurol..

[bib14] Ball G. (2012). The effect of preterm birth on thalamic and cortical development. Cereb. Cortex.

[bib15] Ball G. (2013). Development of cortical microstructure in the preterm human brain. Proc. Natl. Acad. Sci. USA.

[bib16] Bastiani M. (2019). Automated processing pipeline for neonatal diffusion MRI in the developing Human Connectome Project. Neuroimage.

[bib17] Batalle D. (2017). Early development of structural networks and the impact of prematurity on brain connectivity. Neuroimage.

[bib18] Batalle D. (2019). Different patterns of cortical maturation before and after 38 weeks gestational age demonstrated by diffusion MRI in vivo. Neuroimage.

[bib19] Baumann N., Pham-Dinh D. (2001). Biology of oligodendrocyte and myelin in the mammalian central nervous system. Physiol. Rev..

[bib20] Behrens T.E.J. (2003). Characterization and propagation of uncertainty in diffusion-weighted MR imaging. Magn. Reson. Med..

[bib21] Bhutta A.T. (2002). Cognitive and behavioral outcomes of school-aged children who were born preterm - A meta-analysis. Jama-J. Am. Med. Assoc..

[bib22] Buckner R.L., Andrews-Hanna J.R., Schacter D.L. (2008). The brain's default network: anatomy, function, and relevance to disease. Ann. N. Y Acad. Sci..

[bib23] Budde M.D. (2011). The contribution of gliosis to diffusion tensor anisotropy and tractography following traumatic brain injury: validation in the rat using Fourier analysis of stained tissue sections. Brain.

[bib24] Budde M.D., Annese J. (2013). Quantification of anisotropy and fiber orientation in human brain histological sections. Front Integr. Neurosci..

[bib25] Bystron I., Blakemore C., Rakic P. (2008). Development of the human cerebral cortex: Boulder Committee revisited. Nat. Rev. Neurosci..

[bib26] Cameron R.S., Rakic P. (1991). Glial-cell lineage in the cerebral-cortex - a review and synthesis. Glia.

[bib27] Cavanna A.E., Trimble M.R. (2006). The precuneus: a review of its functional anatomy and behavioural correlates. Brain.

[bib28] Chan W.Y., Yew D.T. (1998). Apoptosis and Bcl-2 oncoprotein expression in the human fetal central nervous system. Anat. Rec..

[bib29] Chang L.J. (2013). Decoding the Role of the Insula in Human Cognition: Functional Parcellation and Large-Scale Reverse Inference. Cereb. Cortex.

[bib30] Cismaru A.L. (2016). Altered amygdala development and fear processing in prematurely born infants. Front Neuroanat..

[bib31] Dean D.C. (2017). Mapping white matter microstructure in the one month human brain. Sci. Rep..

[bib32] Dhollander T., Raffelt D., Connelly A. (2016). Unsupervised 3-tissue response function estimation from single-shell or multi-shell diffusion MR data without a co-registered T1 image. ISMRM Workshop Break. Barriers Diffus. MRI.

[bib33] Dimond D. (2020). Early childhood development of white matter fiber density and morphology. Neuroimage.

[bib34] Dubois J. (2008). Asynchrony of the early maturation of white matter bundles in healthy infants: quantitative landmarks revealed noninvasively by diffusion tensor imaging. Hum. Brain Mapp..

[bib35] Dudink J. (2015). Recent advancements in diffusion MRI for investigating cortical development after preterm birth-potential and pitfalls. Frontiers in Human. Neuroscience.

[bib36] Duvanel C.B. (1999). Long-term effects of neonatal hypoglycemia on brain growth and psychomotor development in small-for-gestational-age preterm infants. J. Pediatr..

[bib37] Eaton-Rosen Z. (2015). Longitudinal measurement of the developing grey matter in preterm subjects using multi-modal MRI. Neuroimage.

[bib38] Fair D.A. (2008). The maturing architecture of the brain's default network. Proc. Natl. Acad. Sci. USA.

[bib39] Fischi-Gomez E. (2015). Structural Brain Connectivity in School-Age Preterm Infants Provides Evidence for Impaired Networks Relevant for Higher Order Cognitive Skills and Social Cognition. Cereb. Cortex.

[bib40] Fransson P., Marrelec G. (2008). The precuneus/posterior cingulate cortex plays a pivotal role in the default mode network: Evidence from a partial correlation network analysis. Neuroimage.

[bib41] Gajamange S. (2018). Fibre-specific white matter changes in multiple sclerosis patients with optic neuritis. Neuroimage-Clin..

[bib42] Genc S. (2018). Development of white matter fibre density and morphology over childhood: A longitudinal fixel-based analysis. Neuroimage.

[bib43] Gimenez M. (2006). Abnormal orbitofrontal development due to prematurity. Neurology.

[bib44] Graven S.N., Browne J.V. (2008). Auditory development in the fetus and infant. Newborn Infant Nurs. Rev..

[bib45] Grussu F. (2017). Neurite dispersion: a new marker of multiple sclerosis spinal cord pathology? Annals of Clinical and Translational. Neurology.

[bib46] Gui L. (2019). Longitudinal study of neonatal brain tissue volumes in preterm infants and their ability to predict neurodevelopmental outcome. Neuroimage.

[bib47] He L.X. (2020). Synaptic development of layer V pyramidal neurons in the prenatal human prefrontal neocortex: a Neurolucida-aided Golgi study. Neural Regen. Res..

[bib48] Heidemann R.M. (2010). Diffusion Imaging in Humans at 7T Using Readout-Segmented EPI and GRAPPA. Magn. Reson. Med..

[bib49] Hermoye L. (2006). Pediatric diffusion tensor imaging: normal database and observation of the white matter maturation in early childhood. Neuroimage.

[bib50] Hernandez Fernandez, M., et al., 2017, cuDIMOT: a CUDA toolbox for modelling the brain tissue microstructure from diffusion MRI. In: GPU Technology Conference. Vol., ed.^eds., Munich, germany.

[bib51] Huppi P.S. (1998). Microstructural development of human newborn cerebral white matter assessed in vivo by diffusion tensor magnetic resonance imaging. Pediatr. Res..

[bib52] Huppi P.S. (1998). Quantitative magnetic resonance imaging of brain development in premature and mature newborns. Ann. Neurol..

[bib53] Huttenlocher P.R. (1990). Morphometric study of human cerebral-cortex development. Neuropsychologia.

[bib54] Huttenlocher P.R., Dabholkar A.S. (1997). Regional differences in synaptogenesis in human cerebral cortex. J. Comp. Neurol..

[bib55] Inder T.E. (2005). Abnormal cerebral structure is present at term in premature infants. Pediatrics.

[bib56] Kelly C.E. (2020). Long-term development of white matter fibre density and morphology up to 13 years after preterm birth. medRxiv.

[bib57] Kinney H.C. (1988). Sequence of central nervous system myelination in human infancy. II. Patterns of myelination in autopsied infants. J. Neuropathol. Exp. Neurol..

[bib58] Kiss J.Z., Vasung L., Petrenko V. (2014). Process of cortical network formation and impact of early brain damage. Curr. Opin. Neurol..

[bib59] Kleinnijenhuis M. (2013). Layer-specific diffusion weighted imaging in human primary visual cortex in vitro. Cortex.

[bib60] Knickmeyer R.C. (2008). A structural MRI study of human brain development from birth to 2 years. J. Neurosci..

[bib61] Koelsch S. (2010). Towards a neural basis of music-evoked emotions. Trends Cogn. Sci..

[bib62] Koelsch S. (2014). Brain correlates of music-evoked emotions. Nat. Rev. Neurosci..

[bib63] Koelsch S., Skouras S., Lohmann G. (2018). The auditory cortex hosts network nodes influential for emotion processing: An fMRI study on music-evoked fear and joy. Plos One.

[bib64] Kostovic I. (2014). Perinatal and early postnatal reorganization of the subplate and related cellular compartments in the human cerebral wall as revealed by histological and MRI approaches. Brain Struct. Funct..

[bib65] Kostovic I., Jovanov-Milosevic N. (2006). The development of cerebral connections during the first 20-45 weeks' gestation. Semin. Fetal Neonatal Med..

[bib66] Kostovic I., Judas M. (2010). The development of the subplate and thalamocortical connections in the human foetal brain. Acta Paediatr..

[bib67] Kotilahti K. (2010). Hemodynamic responses to speech and music in newborn infants. Hum. Brain Mapp..

[bib68] Kringelbach M.L. (2005). The human orbitofrontal cortex: Linking reward to hedonic experience. Nat. Rev. Neurosci..

[bib69] Krsnik Z. (2017). Growth of thalamocortical fibers to the somatosensory cortex in the human fetal brain. Front Neurosci..

[bib70] Kunz N. (2014). Assessing white matter microstructure of the newborn with multi-shell diffusion MRI and biophysical compartment models. Neuroimage.

[bib71] Largo R. (1989). Significance of prenatal, perinatal and postnatal factors in the development of AGA preterm infants at five to seven years. Dev. Med. Child Neurol..

[bib72] Lasky R.E., Williams A.L. (2005). The Development of the Auditory System from Conception to Term. NeoReviews.

[bib73] Leech R., Sharp D.J. (2014). The role of the posterior cingulate cortex in cognition and disease. Brain.

[bib74] Lejeune F. (2019). Effects of an early postnatal music intervention on cognitive and emotional development in preterm children at 12 and 24 months: preliminary findings. Front. Psychol..

[bib75] Leuze C.W.U. (2014). Layer-specific intracortical connectivity revealed with diffusion MRI. Cereb. Cortex.

[bib76] Lordier L. (2018). Music processing in preterm and full-term newborns: A psychophysiological interaction (PPI) approach in neonatal fMRI. Neuroimage.

[bib77] Lordier L. (2019). Music in premature infants enhances high-level cognitive brain networks. Proc. Natl. Acad. Sci. USA.

[bib78] Lossi L., Merighi A. (2003). In vivo cellular and molecular mechanisms of neuronal apoptosis in the mammalian CNS. Prog. Neurobiol..

[bib79] Loukas S. (2022). Musical memories in newborns: A resting-state functional connectivity study. Hum. Brain Mapp..

[bib80] Mackes N.K. (2018). Tracking emotions in the brain - Revisiting the Empathic Accuracy Task. Neuroimage.

[bib81] Malhotra A. (2019). Advanced MRI analysis to detect white matter brain injury in growth restricted newborn lambs. Neuroimage-Clin..

[bib82] Marin-Padilla M. (1992). Ontogenesis of the pyramidal cell of the mammalian neocortex and developmental cytoarchitectonics: a unifying theory. J. Comp. Neurol..

[bib83] Mars R.B. (2012). On the relationship between the "default mode network" and the "social brain". Front Hum. Neurosci..

[bib84] Martinet M. (2013). [Development and assessment of a sensory-motor scale for the neonate: a clinical tool at the bedside]. Arch. Pedia.

[bib85] McKinstry R.C. (2002). Radial organization of developing preterm human cerebral cortex revealed by non-invasive water diffusion anisotropy MRI. Cereb. Cortex.

[bib86] Montagna A., Nosarti C. (2016). Socio-Emotional Development Following Very Preterm Birth: Pathways to Psychopathology. Front. Psychol..

[bib87] Mukherjee P. (2002). Diffusion-tensor MR imaging of gray and white matter development during normal human brain maturation. AJNR Am. J. Neuroradiol..

[bib88] Neil J.J. (1998). Normal brain in human newborns: apparent diffusion coefficient and diffusion anisotropy measured by using diffusion tensor MR imaging. Radiology.

[bib89] Nosarti C. (2014). Preterm birth and structural brain alterations in early adulthood. Neuroimage-Clin..

[bib90] Nossin-Manor R. (2013). Quantitative MRI in the very preterm brain: Assessing tissue organization and myelination using magnetization transfer, diffusion tensor and T-1 imaging. Neuroimage.

[bib91] Ohnishi T. (2001). Functional anatomy of musical perception in musicians. Cereb. Cortex.

[bib92] Oishi K. (2010).

[bib93] Olson I.R., Ploaker A., Ezzyat Y. (2007). The Enigmatic temporal pole: a review of findings on social and emotional processing. Brain.

[bib94] Pannek K. (2018). Fixel-based analysis reveals alterations is brain microstructure and macrostructure of preterm-born infants at term equivalent age. Neuroimage-Clin..

[bib95] Pecheva D. (2019). Fixel-based analysis of the preterm brain: Disentangling bundle-specific white matter microstructural and macrostructural changes in relation to clinical risk factors. Neuroimage-Clin..

[bib96] Perani D. (2010). Functional specializations for music processing in the human newborn brain. Proc. Natl. Acad. Sci. USA.

[bib97] Peretz I. (1994). Functional dissociations following bilateral lesions of auditory-cortex. Brain.

[bib98] Peters A., Jones E.G. (1985).

[bib99] Peterson B.S. (2000). Regional brain volume abnormalities and long-term cognitive outcome in preterm infants. Jama-J. Am. Med. Assoc..

[bib100] Pouchelon G., Jabaudon D. (2014). Nurturing the cortex's thalamic nature. Curr. Opin. Neurol..

[bib101] Radley J.J., Morrison J.H. (2005). Repeated stress and structural plasticity in the brain. Ageing Res. Rev..

[bib102] Raffelt D.A. (2015). Connectivity-based fixel enhancement: Whole-brain statistical analysis of diffusion MRI measures in the presence of crossing fibres. Neuroimage.

[bib103] Raffelt D.A. (2017). Investigating white matter fibre density and morphology using fixel-based analysis. Neuroimage.

[bib104] Rakic P. (2003). Developmental and evolutionary adaptations of cortical radial glia. Cereb. Cortex.

[bib105] Rogers C.E. (2012). Regional Cerebral Development at Term Relates to School-Age Social-Emotional Development in Very Preterm Children. J. Am. Acad. Child Adolesc. Psychiatry.

[bib106] Rowley C.D. (2017). Age-Related Mapping of Intracortical Myelin from Late Adolescence to Middle Adulthood Using T-1-Weighted MRI. Hum. Brain Mapp..

[bib107] Sa de Almeida J. (2019). Music enhances structural maturation of emotional processing neural pathways in very preterm infants. Neuroimage.

[bib108] Salami M. (2003). Change of conduction velocity by regional myelination yields constant latency irrespective of distance between thalamus and cortex. Proc. Natl. Acad. Sci. USA.

[bib109] Sarkamo T., Tervaniemi M., Huotilainen M. (2013). Music perception and cognition: development, neural basis, and rehabilitative use of music. Wiley Interdisciplinary Reviews-Cognitive. Science.

[bib110] Shim S.Y. (2012). Altered microstructure of white matter except the corpus callosum is independent of prematurity. Neonatology.

[bib111] Simmons L.E. (2010). Preventing Preterm Birth and Neonatal Mortality: Exploring the Epidemiology, Causes, and Interventions. Semin. Perinatol..

[bib112] Sizonenko S.P.V. (2007). Developmental changes and injury induced disruption of the radial organization of the cortex in the immature rat brain revealed by in vivo diffusion tensor MRI. Cereb. Cortex.

[bib113] Smith R.E. (2013). SIFT: Spherical-deconvolution informed filtering of tractograms. Neuroimage.

[bib114] Smith S.M. (2004). Advances in functional and structural MR image analysis and implementation as FSL. Neuroimage.

[bib115] Smyser C.D. (2010). Longitudinal analysis of neural network development in preterm infants. Cereb. Cortex.

[bib116] Smyser T.A. (2016). Cortical gray and adjacent white matter demonstrate synchronous maturation in very preterm infants. Cereb. Cortex.

[bib117] Spittle A.J. (2009). Early emergence of behavior and social-emotional problems in very preterm infants. J. Am. Acad. Child Adolesc. Psychiatry.

[bib118] Sridharan D., Levitin D.J., Menon V. (2008). A critical role for the right fronto-insular cortex in switching between central-executive and default-mode networks. Proc. Natl. Acad. Sci. USA.

[bib119] Tervaniemi M., Hugdahl K. (2003). Lateralization of auditory-cortex functions. Brain Res Brain Res Rev..

[bib120] Thompson D.K. (2007). Perinatal risk factors altering regional brain structure in the preterm infant. Brain.

[bib121] Thompson D.K. (2013). Hippocampal shape variations at term equivalent age in very preterm infants compared with term controls: perinatal predictors and functional significance at age 7. Neuroimage.

[bib122] Tournier J.D. (2019). MRtrix3: A fast, flexible and open software framework for medical image processing and visualisation. Neuroimage.

[bib123] Tournier, J.-D., Calamante, F., Connelly, A., 2010, Improved probabilistic streamlines tractography by 2nd order integration over fibre orientation distributions. Proc. Intl. Soc. Mag. Reson. Med. (ISMRM). 18.

[bib124] van den Heuvel M.P. (2015). The neonatal connectome during preterm brain development. Cereb. Cortex.

[bib125] Van’t Hooft J. (2015). Predicting developmental outcomes in premature infants by term equivalent MRI: systematic review and meta-analysis. Syst. Rev..

[bib126] Vinall J. (2012). Neonatal pain in relation to postnatal growth in infants born very preterm. Pain.

[bib127] Volpe J.J. (2001).

[bib128] Volpe J.J. (2009). Brain injury in premature infants: a complex amalgam of destructive and developmental disturbances. Lancet Neurol..

[bib129] Wang N. (2019). Neurite orientation dispersion and density imaging of mouse brain microstructure. Brain Struct. Funct..

[bib130] Webb A.R. (2015). Mother's voice and heartbeat sounds elicit auditory plasticity in the human brain before full gestation. Proc. Natl. Acad. Sci. USA.

[bib131] White T.P. (2014). Dysconnectivity of neurocognitive networks at rest in very-preterm born adults. Neuroimage-Clin..

[bib132] Whitfield-Gabrieli S., Ford J.M. (2012). Default mode network activity and connectivity in psychopathology. Annu. Rev. Clin. Psychol..

[bib133] Wildgruber D. (2005). Identification of emotional intonation evaluated by fMRI. Neuroimage.

[bib134] Witt A. (2014). Emotional and effortful control abilities in 42-month-old very preterm and full-term children. Early Hum. Dev..

[bib135] Zamorano A.M. (2017). Insula-based networks in professional musicians: evidence for increased functional connectivity during resting state fMRI. Hum. Brain Mapp..

[bib136] Zatorre R.J., Peretz I., Penhune V. (2009). Neuroscience and Music ("Neuromusic") III: disorders and plasticity. Ann. N. Y. Acad. Sci..

[bib137] Zhang H. (2012). NODDI: Practical in vivo neurite orientation dispersion and density imaging of the human brain. Neuroimage.

