# Supplementary material for: Music impacts brain cortical microstructural maturation in very preterm infants: A longitudinal diffusion MR imaging study
Source: Dev Cogn Neurosci. 2023 May 11;61:101254. doi: 10.1016/j.dcn.2023.101254 (PMC10200857; doi:10.1016/j.dcn.2023.101254)
Supplement: Supplementary file 1 — Supplementary material [file mmc1.docx]

**Supplementary material**

**Supplementary Figures**

Supplementary Fig. S1

Flow chart of the participant inclusion process

Supplementary Fig. S1: 54 very preterm infants (GA at birth <32 weeks) were recruited at the neonatal unit of the University Hospitals of Geneva (HUG), Switzerland, from 2017 to 2020, as part of a prospective randomized clinical trial entitled ‘The effect of music on preterm infant’s development’ (NCT03689725). The 54 infants were randomized into two groups, 28 received a music intervention during NICU stay (PTM) and 26 received standard-of-care during NICU stay (PTC). Randomization was performed using an aleatory function for an unpredictable allocation sequence with concealment of that sequence until assignment occurred. The random allocation, enrolment of patients and assignment of patients to intervention was performed by the research assistant. Parents, music intervention providers, and caregivers were blinded to the group assignment. Exclusion criteria comprised detection of severe brain lesions on MRI, such as intraventricular haemorrhage stage III-IV, hydrocephaly or leukomalacia, microcephaly or macrocephaly and presence of congenital syndrome.

Six preterm infants were excluded from the study due to parental refusal (1 PTM, 4 PTC) and genetic problems (1 PTM). Infants whose MRI protocol acquisition was incomplete (not comprising a T2-weighted image and/or complete multi-shell diffusion imaging (MSDI) sequence), without both longitudinal time-points or whose images presented registration issues during pre-processing, were excluded from the analysis. The final sample of infants used for the analysis consisted of: 40 VPT infants, from which 21 PTM and 19 PTC.

Supplementary Fig. S2

Headphones adapted for preterm infant’s head and preterm infants using the headphones set.

Supplementary Fig. 21: Photos of the headphones specifically designed for the project, adapted to preterm infants’ head size. Headphones set (left top); very preterm infant participating in the intervention using the headphones in the NICU unit (left bottom); photo from a cover of a special issue from National Geographic, which included an article about our study (photo from Craig Cutler).

Supplementary Fig. S3

Final study-specific FOD template

Supplementary Fig. S3: Coronal, axial and sagittal view of the unbiased study-specific FOD template, computed using the 40 intra-subject templates (19 PTC and 21 PTM). The intra-subject templates were generated using the time-point 1 and time-point 2 subjects’ FOD maps, which were rigidly transformed to midway space and then averaged. These intra-subject templates served as input to create the final study-specific population template used for the longitudinal analyses.
